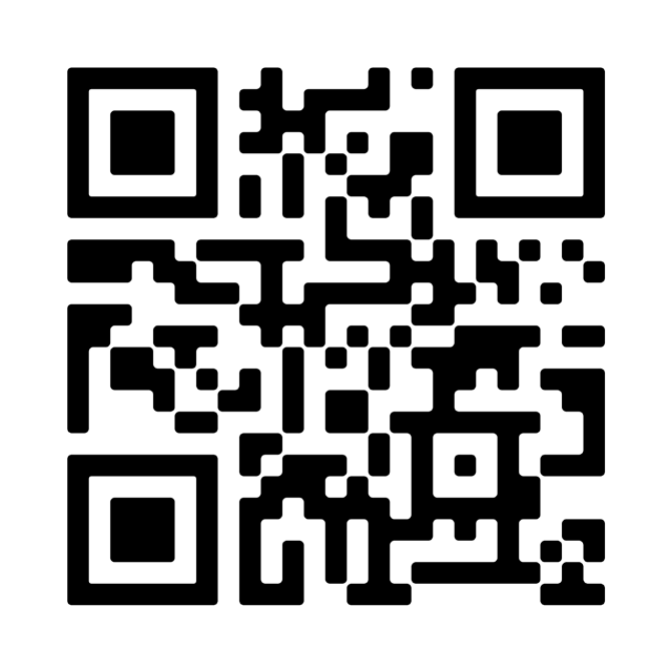# Vaginal power morcellation using a contained bag system: a novel surgical technique

**DOI:** 10.52054/FVVO.16.4.044

**Published:** 2024-12-27

**Authors:** F Fuentes, V Maestri, M Tessmann Zomer Kondo, W Kondo

**Affiliations:** Department of Obstetrics and Gynecology, Hospital of Curico, Curico, Chile; Department of Gynecological Surgery, Vita Batel Hospital, Curitiba, Brazil

**Keywords:** Power morcellation, laparoscopic, endoscopic pouch

## Abstract

**Background:**

To decrease the risk of unsuspected malignancies disseminating, several studies have shown the safety of using a containment bag to limit tissue dissemination during manual or power morcellation. Furthermore, in 2020, the FDA recommended performing laparoscopic power morcellation for myomectomy or hysterectomy only within a tissue containment system.

**Objective:**

To show step-by-step a new surgical technique using vaginal power morcellation within an endoscopic pouch without adding or extending other incisions.

**Materials and methods:**

Video article describing vaginal power morcellation.

**Results:**

To perform power morcellation through vaginal introit, additional operative time was only 13 minutes.

**Conclusions:**

Vaginal power morcellation using a contained bag system is technically feasible and efficient. Furthermore, it may prevent intraperitoneal dissemination of tissue fragments whilst minimising the need for additional surgical incisions.

## Learning Objective

Morcellation is a helpful procedure to reduce and fragment the size of specimens and remove them via small openings in the body without needing a laparotomy. To date, several techniques for morcellation, either with a scalpel or electromechanical energy, have been described. The specimen can be extracted either vaginally, with a mini-laparotomy, or through an abdominal 15 mm port.

This video shows step-by-step how to perform power morcellation using an endoscopic containment pouch through vaginal introit, without adding or extending other incisions.

## Introduction

Advances in surgical techniques have allowed laparoscopic hysterectomies to be performed for increasing larger uteri. However, this approach has resulted in the need for specimen reduction by morcellation (Favero et al., 2012; Foley et al., 2020) for tissue retrieval. Nevertheless, this procedure has a significant limitation: the risk of unsuspected malignancies disseminating, particularly leiomyosarcoma. Several studies have shown the safety of using a containment bag to decrease this risk to limit tissue dissemination during manual or power morcellation (Srouji et al., 2015; Zapardiel et al., 2021). Furthermore, in 2020, the FDA recommended performing laparoscopic power morcellation for myomectomy or hysterectomy only within a tissue containment system (Foley et al., 2020; ACOG, 2019; Zapardiel et al., 2021).

## Patient

A 33-year-old patient, gravida 2, with a history of two caesarean sections, presented a history of abnormal uterine bleeding and chronic pelvic pain that did not respond to medical treatment. Ultrasound imaging demonstrated a 13 x 9.5 x 13cm intramural fibroid (FIGO 2-5) arising from the anterior wall, with an overall uterine volume of 1259 cm^3^.

## Intervention

A laparoscopic hysterectomy with extraction of the specimen through the vagina in a contained bag was performed. Key steps were: (1) Once the laparoscopic hysterectomy was performed, a pouch is placed into the abdominal cavity through the vaginal vault; (2) The bag is unfolded, and the specimen is placed inside; (4) The edges of the bag are exteriorised through the vaginal introit and the umbilical trocar; (5) A 15 mm trocar is placed through the vaginal introit; (6) A pseudo- pneumoperitoneum is established using the umbilical trocar, reaching 12 mm mercury; (7) Under direct vision, power morcellation is performed vaginally; (8) Finally, withdrawing the bag through the vaginal is performed.

## Discussion and conclusion

Laparoscopic hysterectomy has many benefits over open surgery, including lower blood loss, shorter hospital stays, decreased pain, and faster postoperative recovery (Schindlbeck et al., 2008). Advances in surgical techniques have allowed laparoscopic surgery even for large uteri. However, this approach has implicated the need for specimen reduction by morcellation (Favero et al., 2012; Foley et al., 2020). Nevertheless, this procedure has a major limitation: the risk of disseminating unsuspected malignancies, particularly leiomyosarcoma. Because of this concern, in April 2014, the FDA issued a safety communication discouraging the use of laparoscopic power morcellation (Foley et al., 2020; Wood et al., 2018).

To decrease the risk of dissemination, several studies have shown the safety of using a containment bag to limit tissue dissemination during manual or power morcellation (Srouji et al., 2015; Zapardiel et al., 2021; Cohen et al., 2014). Other studies on contained power morcellation demonstrated feasibility with either a single-port or multiport approach (Srouji et al., 2015; Tissue Extraction Task Force Members, 2018; Zapardiel et al., 2021).

In December 2020, the FDA issued the final guidance related to laparoscopic power morcellators. They recommended performing laparoscopic power morcellation for myomectomy or hysterectomy only with a tissue containment system and in appropriately selected patients (ACOG, 2019).

To date, several techniques for morcellation, either with a scalpel or electromechanical energy, have been described. The specimen can be extracted either vaginal introit, a mini-laparotomy, or through an abdominal 15 mm port (Favero et al., 2012; Foley et al., 2020; Marchand et al., 2021). Contained power morcellation offers the efficiency and speed of power morcellation while utilising a containment system helping to prevent intraabdominal dissemination of tissue particles (Vargas et al., 2015; Winner et al., 2015; Tissue Extraction Task Force Members, 2018; Zapardiel et al., 2021).

Comparisons across the various modalities of contained tissue extraction have demonstrated similar perioperative outcomes and low risk of complications, with some variability in terms of relative operative time (Favero et al., 2012; Srouji et al., 2015; Tissue Extraction Task Force Members, 2018; Raimondo et al., 2024). Vaginal morcellation prevents the extension of the abdominal incisions, which contributes to cosmesis and hernia risk. Furthermore, carrying out power morcellation appears to decrease the risk of injury to surrounding structures, including the bladder, rectum, and vagina, compared to manual (Foley et al., 2020; Marchand et al., 2021).

Although vaginal power morcellation may be associated with reduced surgical time and complication rates, it should only be performed by an experienced surgeon. In cases involving a large uterus, the direct view of the surgeon may be limited when the specimen is drawn towards the morcellator blade, potentially increasing the risk of injury to adjacent organs. Additionally, the surgeon must thoroughly understand the technique, as operating with a frontal camera view can compromise orientation and movement precision, thereby heightening the risk of complications or rupture of the containment bag.

## Video scan (read QR)

https://vimeo.com/992069429/7c26e241ab?share=copy

**Figure:**